# Gender Differences in Respiratory Morbidity and Mortality of Preterm Neonates

**DOI:** 10.3389/fped.2017.00006

**Published:** 2017-01-30

**Authors:** Courtney Denise Townsel, Sawyer F. Emmer, Winston A. Campbell, Naveed Hussain

**Affiliations:** ^1^Department of Obstetrics and Gynecology, University of Connecticut, Farmington, CT, USA; ^2^Department of Neonatology, Connecticut Children’s Medical Center, Farmington, CT, USA

**Keywords:** preterm, sex, gender, mortality, respiratory morbidity

## Abstract

For the past century, researchers have underscored the “disadvantage” observed in respiratory morbidity and mortality of male newborns. In this contemporary review, we examine gender differences in preterm infant respiratory morbidity and mortality specifically appraising differences in the very low birth weight (VLBW) population as well as the late preterm (LPT) population. In the era of postnatal surfactant and antenatal corticosteroids, the gender gap in neonatal outcomes has not narrowed. Structural, physiologic, and hormonal sex differences may be at the root of this disparity. Further exploration into the origin of gender differences in respiratory morbidity and neonatal mortality will shape future therapies. These therapies may need to be gender specific to close the gender gap.

## Introduction

Disparities in neonatal outcomes by demographics such as race, birthweight, and gender have been well documented since the early twentieth century (1, 2). In addition to the non-modifiable demographics mentioned, gestational age remains the key factor tightly correlated with neonatal and infant morbidity and mortality. In 2014, 9.6% of all births occurred before 37 weeks, during the preterm period, in the United States (3). Infants born prematurely are more likely to suffer major morbidity such as respiratory distress, necrotizing enterocolitis, interventricular hemorrhage (IVH), and experience higher mortality than infants born at term (4). Among these morbidities, the greatest contributor to infant mortality is respiratory disease. The incidence of respiratory distress syndrome (RDS) and subsequent bronchopulmonary dysplasia (BPD) or chronic lung disease (CLD) is higher in infants born prematurely due to insufficient surfactant production by type II pneumocytes.

Twenty-five years ago, synthetic surfactant became widely available for postnatal administration following the landmark study by Fujiwara showing improved survival in artificial surfactant-exposed neonates and subsequent studies supporting its efficacy (5–7). This was followed shortly by a National Institute of Health (NIH) consensus statement published in 1994 supporting the use of antenatal corticosteroids to reduce the risk of RDS in preterm infants between 24 and 34 weeks at risk for preterm delivery (8). In this consensus statement, use of antenatal corticosteroids was associated with reduced rates of RDS, IVH, and overall increase in survival in exposed neonates. In recent years, attention has been shifted to the adverse outcomes associated with delivery in the late preterm (LPT) period, delivery between 34 and 36 completed weeks, and the disproportionate morbidity and mortality they face compared to term infants (9, 10). Underscoring this was a recent randomized controlled trial illustrating the benefits of antenatal corticosteroids for respiratory outcomes in this population (11).

Very low birth weight (VLBW) infants, birth weight less than 1,500 g, account for only 1% of the preterm population but represent greater than 80% of the morbidity and mortality attributed to infants born preterm (3). On the other end of the spectrum, LPT infants are the fastest growing subgroup of neonates and account for 70% of all preterm births (3). In this contemporary review, we will examine gender differences in preterm infant respiratory morbidity and mortality with hopes to shed light on whether the gender disparity gap has narrowed over the past two and a half decades. Within the preterm population, we will specifically examine differences in respiratory morbidity between genders in the VLBW population and the LPT population. This will be followed by an assessment of gender differences in mortality for preterm neonates with emphasis on the VLBW and LPT populations. Finally, we will seek to explore physiologic and hormonal reasons why this gender disparity exists. Understanding the origin of gender differences in respiratory morbidity and overall mortality among male and female preterm infants may lead to more targeted therapies and streamlined interventions to lessen this inequality in the future.

To complete this review, the authors used the Scopus database and searched using the terms *gender, sex, differences, preterm, very low birth weight, late preterm, respiratory morbidity*, and *mortality*. Abstracts and results were reviewed by two authors independently. Manuscripts were excluded if results were not stratified by sex or gender, if the population studied did not include preterm infants and if the text was not in English. There were no geographic restrictions for publication. Gender refers to the socially constructed characteristics of men and women, while sex refers to the genetic makeup and reproductive organs of an individual. For the purposes of this review, the terms *sex* and *gender* are used interchangeably and are based on clinical exam.

## Gender Differences in Preterm Respiratory Morbidity

Since the sentinel study by Wyllie in 1933, many researchers have underscored the “disadvantage” observed in the overall morbidity and mortality of male newborns (1). Nowhere is this difference more evident than in the respiratory morbidity of preterm male infants. An ongoing theme throughout the literature is that males tend to develop RDS and CLD at a much higher rate than their female counterparts. A meta-analysis by Liptzin et al. including data from over 500,000 preterm newborn infants highlighted a sex ratio of 1.56–1.84 (*p* < 0.05) in favor of males for respiratory distress as well as a sex ratio ranging from 1.22 to 1.38 (*p* < 0.05) again favoring males for CLD compared to females (12–15). Males in this study also had increased rates of lower respiratory tract infection such as bronchiolitis and pneumonia (sex ratio male:female 1.43 and 1.49, respectively), despite similar infection rates between sexes.

### VLBW Less Than 1,500 g and Gender Differences in Respiratory Morbidity

Over the past decade, several studies have reported on the respiratory morbidity in the most at-risk preterm group, VLBW neonates. In a study of 236 infants less than 29 weeks gestation 36.2% of males compared to 19.2% of females developed CLD (*p* = 0.004) (16). Short-term respiratory morbidity was also worse in males as they required mechanical ventilation at higher rates within the first 6 h of life (60.8 versus 46.2%, *p* = 0.026) and were found to have lower mean arterial pressures (10.5 versus 11.6 mmHg, *p* = 0.042) within the first day of life (16). In a subgroup analysis of 175 extremely low birth weight infants, birth weight less than 1,000 g, males were intubated longer than females (6 versus 4 days, *p* = 0.101) and were dosed more frequently (2 doses versus 1 dose, *p* = 0.055), particularly in the first 6 h of life (66.3 versus 47.2%, *p* = 0.011). However, some of these differences did not reach statistical significance. Stevenson et al. underscored this significant difference between the sexes in regards to pulmonary morbidity in his report of over 6,700 VLBW infants where males had higher rates of RDS (OR 1.41, CI 1.28–1.56), supplemental oxygen usage (OR 1.32, CI 1.14–1.54), required higher rates of ventilator support (OR 1.34, CI 1.20–1.50), surfactant use (OR 1.33, CI 1.10–1.60), and developed pneumothorax at a higher rate (OR 1.33, CI 1.09–1.62) (17). While significant differences were observed in this study, it is also important to note that males received antenatal corticosteroids at a lower rate than females (20 versus 24%, OR 0.80, CI 0.71–0.90) and that overall antenatal corticosteroid administration was low, which may be a function of the study period (1991–1993) occurring before the 1994 NIH consensus statement on antenatal corticosteroids in preterm deliveries.

The findings of Brothwood et al. were very similar to the findings of Stevenson et al. In this study the rates of RDS, supplemental oxygen usage and need for mechanical ventilation were higher for males than females. This study, unlike many studies investigating this population, followed this VLBW cohort for 2 years and found that early neonatal respiratory morbidity in males was associated with higher rates of neurodevelopmental abnormalities (67% in males versus 33% in females, *p* = 0.028) including cerebral palsy, sensorineural hearing loss, retinopathy, and retardation (18). Also at 2 years of life, males were found to have lower hearing and speech scores (94.9 in males versus 107.3 in females, *p* < 0.001) and worse personal social skills scores (103.6 versus 116.9, *p* < 0.05) compared to females (18). While prematurity may be a major contributing factor to long-term neurodevelopmental outcomes, it is also important to understand that other factors may also play a vital role in cognitive and emotional responses in early childhood. The impact of the child’s environment and postnatal exposures play a large role and were not directly addressed in the prior study. Caution must be taken when attributing association in this context.

### LPT and Gender Differences in Respiratory Morbidity

Although earlier gestational ages have been linked to worsened respiratory outcomes for decades, there are recent data that suggest male sex, regardless of gestational age, increases the risk of adverse respiratory outcomes in newborns. Anadkat et al. reviewed the impact of male sex on the frequency of RDS in infants born between 34 and 42 weeks and demonstrated that males were at significantly increased risk of respiratory morbidity in the LPT period (34 weeks OR 114.1, 35 weeks OR 41.0, and 36 weeks OR 15.4: overall OR 1.68; *p* < 0.001 for all values) (14). The authors attributed this disparity to hormonal regulation differences between the sexes while developing *in utero*. Although hormonal regulation may be one influencer causing this drastic difference in respiratory morbidity, the interplay of other factors is crucial to understand the divergent physiology of lung development between sexes.

These findings are supported by the work of Altman et al. who investigated risk factors for moderately preterm (30–34 weeks), LPT (35–36 weeks), and term infants (37–41 weeks, reference). LPT infants were at increased risk for transient tachypnea of the newborn (TTN) (OR 9.5, CI 8.6–10.5) and RDS (OR 38.7, CI 31.6–47.3) compared to term infants (19). Male gender was found to be a risk factor for poor respiratory outcome in this cohort (TTN OR 1.25, CI 1.03–1.52 and RDS OR 1.58, CI 1.28–1.96).

## Gender Differences in Preterm Mortality

Preterm neonates account for the majority of neonatal morbidity and mortality. The major morbidity contributing to mortality in this cohort is respiratory insult. The gender differences among preterm infants in respiratory morbidity were highlighted above. As previously mentioned, the use of postnatal synthetic surfactant gained widespread support in the early 1990s. During the timeframe where general use of postnatal surfactant was being established de Kleine compared neonatal mortality, death within the first 28 days of life, between infants born in 1983 and those born in 1993. Neonatal mortality decreased from 52.1 to 31.8% in VLBW infants and from 15.2 to 11.3% in infants delivered between 28 and 31 weeks. The effect of male gender on mortality increased significantly during the study period with gender having no statistically significant effect in 1983 (OR 1.35, CI 0.92–1.99) to male sex contributing significantly in 1993 (OR 2.52, CI 1.42–4.55) (20). Postnatal surfactant alone improved overall mortality, but the gender gap in survival remained. Use of antenatal corticosteroids gained momentum in the mid-1990s shortly after this study. We will examine the impact of both antenatal corticosteroids and postnatal surfactant use in our two target populations.

### VLBW Less Than 1,500 g and Gender Differences in Mortality

A study by Jones et al. of over 19,000 Canadian preterm neonates at 17 different centers from 1996 to 1997 showed survival to discharge was higher in VLBW females (88.9 versus 85.6%, *p* < 0.05) (21). One criticism of this data was the difference in antenatal corticosteroid administration across genders, 69.4% in females versus 66.2% in males (*p* < 0.05). Lower survival and less frequent antenatal corticosteroid use in males directly reflected the higher rate of CLD found in this group (25.7% males versus 18.9% females, *p* < 0.05). This study captured outcomes in the immediate period following widespread support for use of antenatal corticosteroids. A subsequent study of over 2,700 Canadian neonates born between 24 and 26 weeks from 2000 to 2005 supported the previous findings by Jones showing mortality was higher in the male cohort (OR 0.78, CI 0.62–0.99) (22). The authors of this study concluded that males delivered between 24 and 26 weeks in the post-surfactant era remain at higher risk of mortality than girls despite equal exposure to antenatal corticosteroids. To further investigate the effect of antenatal corticosteroid use on neonatal mortality, Fanaroff compared neonatal death in VLBW infants in the immediate postnatal–antenatal steroid era (1995–1996) to the outcomes of neonates over the next 6 years (1997–2002). There was no significant increase in survival without neonatal and long-term morbidity between the two cohorts. Females in both groups had higher survival rates with observed functional maturity superior to gestational age and birthweight matched males (23).

### LPT and Gender Differences in Mortality

Recently, there has been a focus on the LPT neonate and the disparity in mortality this cohort faces compared to infants born at term. In a study of 252 LPT infants, mortality was higher for this cohort compared to term infants (2.3 versus 0%, *p* = 0.04) with male gender highlighted as an independent risk factor in this cohort (24). Given the low rate of mortality in the LPT group compared to neonates born at less than 34 weeks, many studies have chosen to highlight the primary contributor to mortality in LPT infants, respiratory morbidity, as opposed to mortality. In 2016, the authors of a prospective randomized controlled trial demonstrated a 20% reduction in their composite primary outcome that included need for CPAP, supplemental oxygen, and mechanical ventilation for LPT infants given betamethasone (*p* = 0.02) (11). These LPT infants were also found to have lower rates of transient TTN (*p* = 0.002), required less surfactant (*p* = 0.03), and had lower rates of BPD (*p* = 0.04). This study was not powered to show a difference in mortality and furthermore did not provide results stratified by gender. With the new recommendation of antenatal corticosteroids for this LPT group, further investigation is needed to determine whether the male sex contribution to mortality risk in LPT infants persists with steroid administration.

## Exploring the Origins of Gender Differences in Neonatal Respiratory Morbidity and Mortality

The apparent male disadvantage in morbidity and mortality has again been highlighted in this review. We present the clinical evidence from the past three decades that continues to support a sex disparity despite the advent of antenatal corticosteroid administration and postnatal surfactant use. From a basic macroscopic level, male fetuses tend to weigh more at any given gestational age and thus tend to have more alveoli and alveolar surface area than gestational age-matched females (22, 25, 26). Surfactant production, however, has been shown to appear earlier in female lung development than in males (27). This earlier presence of surfactant seems to prevent the early closure of female alveoli and small airways, which may contribute to the higher airflow and decreased resistance found in the female respiratory system. Fleisher et al. showed that both the 2:1 lecithin/sphingomyelin (L/S) ratio and the appearance of phosphatidylglycerol, a component of surfactant, occurred a week earlier in females than males (27).

As mentioned before, antenatal corticosteroid administration reduces respiratory morbidity and thus decreases neonatal mortality. Corticosteroids have been shown to upregulate airway Na^+^–K^+^ pump activity which facilitates shunting of fluid out of the fetal lung (28). Uncleared fluid within the fetal lung predisposes neonates to RDS. However, despite equal administration of antenatal corticosteroids, we continue to see RDS at a higher rate in males. This may be explained by the fact that male neonates have lower rates of alveolar sodium transport channels than females (29). A decrease in sodium transport channels may contribute to fluid accumulation in the lung which would hinder gas exchange and increase the risk of RDS.

Sex hormones have also been shown to have an effect on the development of the fetal lung. When analyzing the components of fetal lung tissue, androgen receptors are found in higher density on the surface of the epithelial cells that modulate bronchiole budding in the early fetal lung (30). These same cells express 5-alpha reductase in their cytoplasm in high amounts which may indicate dihydrotestosterone, and other androgens have an effect on the initial development of the bronchiole tree (30). Androgens have also been shown to inhibit surfactant production by altering epidermal growth factor and transforming growth factor β1 (31). Higher levels of androgens decrease the activity of these two transcription factors in type II alveolar cells downregulating production of surfactant. One animal study showed that female rabbits given androgens *in utero* had delayed lung development while mothers with male offspring given antiandrogens showed no sex difference in surfactant production compared to normal females (25). The male disadvantage is not simply a consequence of surfactant insufficiency but is also driven by the negative effect exhibited by androgens on alveolar cells.

**Figure 1:**
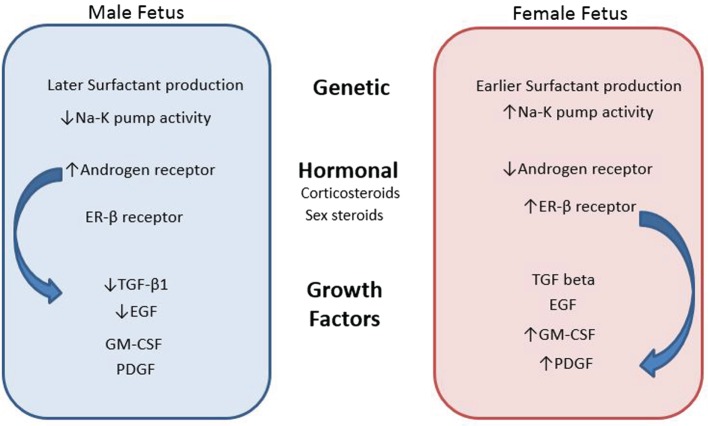
**Contributing factors to gender differences in neonatal respiratory morbidity and mortality**.

Alternatively, estrogen has been shown to also influence lung development (32). This mechanism has been postulated to occur through the platelet derived growth factor and granulocyte-macrophage colony-stimulating factor signaling pathways which cause defects in alveolar structure, lung elasticity, and surfactant production (33). Deletion of estrogen receptor β in female mice causes an increase in alveolar size and a decrease in alveolar surface area making these mice more like their male counterparts. This difference can strongly impact the risk of developing RDS in preterm neonates as the decreased surfactant and recoil of the lung contributes to a lower probability of gas exchange in alveoli. The constellation of gender differences described is illustrated in Figure 1.

### Contributing Factors to Gender Differences in Preterm Neonatal Respiratory Morbidity and Mortality

Figure 1 describes the genetic, hormonal, and functional differences between genders that may explain the disparity in respiratory morbidity and mortality between males and females.

## Conclusion

For the past century, researchers have underscored the “male disadvantage” observed in respiratory morbidity and mortality of newborns. Through this contemporary review, we have examined gender differences in preterm infant respiratory morbidity and mortality specifically appraising differences in the VLBW population as well as the LPT population. In the era of postnatal surfactant and widespread use of antenatal corticosteroids, the gender gap in neonatal outcomes has not narrowed. We continue to see stark differences between males and females in regards to acute and CLD as well as mortality. Sex differences in physiology, hormones, and growth factors are fundamentally at the root of this disparity. With this knowledge, new endeavors for gender-specific therapies must be undertaken to narrow this gender gap. Similar to the many other disparities in medicine, the problem of gender differences in neonatal outcomes has been well identified and described, and yet we are no closer to an answer or resolution. Future research should focus on (1) exploring gender-specific therapies to reduce respiratory morbidity in males and (2) further characterizing the effect of antenatal steroids in the LPT infant and how this impacts survival.

## Author Contributions

CT performed a literary search, reviewed articles for inclusion, designed the layout and scope of the review article, and drafted, updated, and organized the review. SE performed a literature search and helped to draft the manuscript. WC helped with defining the scope of the manuscript and organizing the sections for discussion and made large contributions to the editing of this manuscript. NH helped to define the review focus and assisted with edits of the manuscript, organized and created the figure within the manuscript, which outlines the various contributing factors to gender differences in neonatal respiratory morbidity and mortality, and also made large contributions to editing this manuscript.

## Conflict of Interest Statement

The authors declare that the research was conducted in the absence of any commercial or financial relationships that could be construed as a potential conflict of interest.
